# 

**Published:** 1903-01-10

**Authors:** 


					the hospital. "The standard of highest purity."?Lcmcst. Jan. ioth, 1903.
CADBURY'S ww CADBURY'S
COCOA HL Jo mr COCOA
ABSOLUTELY PURE. )9*, <^> ~Sw/ NO DRUQS OR ALKALIES USED.
Ko. 850. Vol. XXXIII. [fgpape"] Saturday,
Jan. 10th, 1903.
[ bS ] prich twopence.

				

